# Transcriptome Profiling Identifies Plant Hormone Signaling Pathway-Related Genes and Transcription Factors in the Drought and Re-Watering Response of *Ginkgo biloba*

**DOI:** 10.3390/plants13192685

**Published:** 2024-09-25

**Authors:** Meiling Ming, Juan Zhang, Jiamin Zhang, Jing Tang, Fangfang Fu, Fuliang Cao

**Affiliations:** State Key Laboratory of Tree Genetics and Breeding, Co-Innovation Center for Sustainable Forestry in Southern China, Nanjing Forestry University, Nanjing 210037, China; mingmeiling@njfu.edu.cn (M.M.); juanzhang@njfu.edu.cn (J.Z.); 2210110624@njfu.edu.cn (J.Z.); tangjing.t@njfu.edu.cn (J.T.)

**Keywords:** drought stress, re-watering, *Ginkgo biloba*, oxidative stress, plant hormone signal transduction, transcription factors

## Abstract

*Ginkgo biloba*, usually referred to as a “living fossil,” is widely planted in many countries because of its medicinal value and beautiful appearance. Owing to ginkgo’s high resistance to drought stress, ginkgo seedlings can even survive withholding water for several days without exhibiting leaf wilting and desiccation. To assess the physiological and transcriptomic mechanisms involved in the drought stress and re-watering responses of *Ginkgo biloba*, ginkgo seedlings were subjected to drought treatment for 15 d (D_15 d) and 22 d (D_22 d) until they had severely wilted, followed by re-watering for 3 d (D_Re3 d) to restore normal growth. Variations in physiological characteristics (relative water content, malondialdehyde (MDA) content, stomatal aperture, and antioxidant enzyme activity) during drought and re-watering were assessed. In total, 1692, 2031, and 1038 differentially expressed genes (DEGs) were upregulated, while 1691, 2820, and 1910 were downregulated in D_15 d, D_22 d, and D_Re3 d, respectively, relative to the control. Three pathways, namely, plant hormone signal transduction, plant–pathogen interaction, and the plant MAPK signaling pathway, were enriched during drought stress and re-watering. The DEGs involved in plant hormone signal transduction pathways (those of IAA, CTK, GA, ABA, ETH, BR, SA, and JA) and the major differentially expressed transcription factors (TFs; *MYB*, *bHLH*, *AP2/ERF*, *NAC*, *WRKY*, and *bZIP*) were identified. Quantitative real-time PCR revealed six TFs as positive or negative regulators of drought stress response. These phenotype-related physiological characteristics, DEGs, pathways, and TFs provide valuable insights into the drought stress and re-watering responses in *G. biloba*.

## 1. Introduction

Drought is a common and serious problem in plant production worldwide, which is expected to worsen with anticipated changes to the climate. Drought stress limits plant growth and distribution by altering metabolic activities as well as physiological and biochemical functions, ultimately damaging plant quality and production [1]. The primary phenotypic changes seen in plants under drought stress are wilting, desiccation, and senescence, which may be caused by various physiological and biochemical responses at the cellular and organismal levels, such as reduced cellular water content, decreased CO_2_ assimilation, membrane damage, stomatal closure, accumulation of reactive oxygen species (ROS), and inhibition of enzyme activity [2].

During drought stress, plants experience an imbalance between electron excitation and the utilization of energy in photosynthesis, caused by decreased carbon assimilation, which leads to the production of ROS, mainly superoxide anion (O_2_^−^) and hydrogen peroxide (H_2_O_2_) [3]. Excess accumulation of ROS results in oxidative stress, the extent of which is usually indicated by intercellular malondialdehyde (MDA) content, which damages cell membranes, DNA, and proteins, ultimately leading to cell death [2]. Plants possess both enzymatic and non-enzymatic mechanisms for scavenging excess ROS. These enzymatic mechanisms are referred to as the enzymatic plant antioxidant defense system, which mainly comprises superoxide dismutase (SOD), catalase (CAT), and peroxidase (POD) [4]. The SOD catalyzes the conversion of O_2_^−^ to the less reactive H_2_O_2_, which is further detoxified to O_2_ and H_2_O through the activities of CAT and POD, ultimately resulting in low intracellular levels of O_2_^−^ and H_2_O_2_ [5].

In contrast, the non-enzymatic system comprises abscisic acid (ABA), tocopherol, carotenoids, glutathione, phenolics, and flavonoids. ABA is considered the principal phytohormone that confers drought tolerance in plants through many morphophysiological and molecular processes, including stomatal adjustment, root development and elongation, and initiation of the ABA-dependent pathway [6]. PYR/PYLs (pyrabactin resistance/PYR-like) are ABA receptors that are necessary for ABA response and transduction. Fourteen genes encode PYR/PYL receptors in Arabidopsis, whereas 11 genes encode PYR/PYL receptors in rice [7]. When plants cope with drought stress, the cellular ABA content increases and ABA binds to PYR/PYLs, which in turn bind and inactivate PP2Cs (protein phosphatase 2C). SnRK2s (serine/threonine-protein kinase 2) are protein kinases that promote ABA responses by auto-activation and phosphorylation of downstream targets and improve ABA-induced physiological, biochemical, and molecular responses, including germination, stomatal movement, root development, and photosynthesis [6]. Moreover, auxins (IAA), cytokinins (CTK), gibberellin (GA), ethylene (ETH), brassinosteroids (BR), salicylic acid (SA), and jasmonic acid (JA) have been reported as crucial phytohormones for managing drought stress [8]. For instance, reduced IAA levels under drought stress conditions can increase the ABA content in plants to improve growth modulation by auxins. AUX/IAA proteins are a large family of auxin co-receptors and transcriptional repressors. Auxin response factors (ARFs) bind directly to the promoters of auxin-responsive genes, allowing them to be transcriptionally activated or repressed and enhancing stress tolerance in plants.

Transcription factors (TFs), such as WRKY [9], MYB (v-myb avian myeloblastosis viral oncogene homolog) [10], AP2/ERF (APETALA2/ethylene-responsive factor) [11], bHLH (basic helix-loop-helix) [12], NAC (NAM, ATAF1/2, and CUC) [13], and bZIP (basic leucine zipper) [14], play important roles in plant response to drought stress via protein interactions and transcriptional regulation of target genes. Numerous studies have demonstrated the crucial biological functions and molecular mechanisms of WRKY TFs in plant responses to various abiotic stressors, including drought stress [9]. Many WRKYs, such as *AtWRKY1* [15], *AtWRKY45* [16], *AtWRKY46*, *AtWRKY54*, and *AtWRKY70* [17] in *Arabidopsis*; *MdWRKY70L* and *MdWRKY115* in apple [18,19]; *PbrWRKY53* [20] in pear, and *FcWRKY70* [21] in *Fortunella crassifolia*, have been identified as positive or negative regulators of drought stress through their binding to the promoters of drought stress-responsive genes. In addition to WRKY, genome-wide transcriptome and expression analyses have shown that many MYB genes are responsive to drought in *Arabidopsis thaliana*, *Zea mays*, *Malus pumila*, and *Populus euphratica* [10]. For example, *AtMYB60*, a regulator of stomatal movement, is downregulated by drought stress, while overexpression of *AtMYB60* leads to hypersensitivity to water stress [22]. The dehydration-responsive element-binding (DREB) protein subfamily members of AP2/ERF are the main regulators of drought stress in both the ABA-independent and ABA-dependent pathways. For instance, *Arabidopsis* DREB2A plays a dual role in water and heat shock stress responses [23]. bHLH TFs are mainly involved in drought stress via the ABA or JA signaling pathways. For example, *AtbHLH006/17/32/92/122/128/130* and *AtMYC2* directly or indirectly regulate the expression of genes involved in the ABA signaling pathway to improve drought resistance in *Arabidopsis* [24]. Similarly, the overexpression of root-specific NAC TFs (*OsNAC5/6/9/10*) in rice changed the root architecture and conferred drought tolerance [25]. Recent studies have revealed the roles of group A bZIP TFs, such as *AREB1/2* and *ABF1/2/3/4* in *Arabidopsis thaliana* and *OsbZIP2/23/42/46*, *OsABF1*, and *OsABI5* in rice, which positively regulate ABA-dependent gene expression [25]. Other groups of bZIP TFs, such as *TGA*, positively regulate SA-dependent gene expression.

Many studies have focused on the physiological and biochemical characteristics, such as changes in photosynthesis, stomatal movement, oxidative stress, antioxidant systems, and secondary metabolites of plants, including ginkgo, in response to drought stress [2,3,26]. Research on the physiological, biochemical, and gene expression changes during drought recovery is limited. *Ginkgo biloba*, usually referred to as a “living fossil,” is widely planted in many countries because of its medicinal value and beautiful appearance. Owing to ginkgo’s high resistance to various biotic and abiotic stresses, especially drought stress, ginkgo seedlings can even survive withholding water or continuous irrigation with 20% PEG6000 solution for several days without exhibiting stress phenotypes such as leaf wilting and desiccation. Given this, we are interested in defining the physiological and biochemical responses of ginkgo and investigating its underlying regulatory mechanisms contributing to drought tolerance through long-term (several days instead of several hours) drought treatments. Therefore, in the current study, the phenotype-related physiological and biochemical responses, gene regulatory mechanisms, and transcriptional reconfiguration of *G. biloba* in response to drought stress and re-watering were investigated by subjecting ginkgo seedlings to drought stress for 22 d, resulting in an obvious leaf wilting phenotype. This study provides new insights into the physiological responses, plant hormone transduction pathways, and key genes that regulate drought stress and re-watering in *G. biloba*.

## 2. Materials and Methods

### 2.1. Plant Materials, Drought, and Re-Watering Treatments

*G. biloba* seedlings were grown in 500 mm × 190 mm × 150 mm nursery pots with a 1:1 *v*/*v* mixture of soil (Xingxing Xiangnong cultivation soil, Jiangsu Xingnong Substrate Technology Co., Ltd., Changshu, China) and vermiculite in a plant growth chamber at a temperature of 25 °C with a 16 h light/8 h dark photoperiod. They were irrigated with running water regularly (once a week) before drought treatment. Thirty-five-day-old seedlings with similar, favorable growth performance were subjected to drought treatment by stopping irrigation and withholding any other water sources. Seedlings in the control group (CK) were irrigated regularly. After 22 d, both the drought and CK seedlings were simultaneously rewatered with sufficient water (Figure S1). Four sampling points were selected for the drought treatment: drought for 0 d (D_0 d, before treatment), drought for 15 d (D_15 d), drought for 22 d (D_22 d), and 3 d after re-watering (D_Re3 d). Four sampling points were also collected for CK: CK for 0 d (CK_0 d, before treatment), CK for 15 d (CK_15 d), CK for 22 d (CK_22 d), and 3 d after re-watering (CK_Re3 d). Six seedlings were harvested randomly at each time point (D_0 d, D_15 d, D_22 d, D_Re3 d, CK_0 d, CK_15 d, CK_22 d, and CK_Re3 d), and the harvested mature leaves from each plant were mixed and frozen immediately in liquid nitrogen and stored at −80 °C until further analysis.

### 2.2. Measurements of Leaf Relative Water Content (RWC), MDA Content, and Antioxidant Enzyme (POD, SOD, and CAT) Activity

Leaf RWC was measured according to the standard method proposed by Barrs and Weatherley [27], with some modifications. The fresh weight (FW) of leaves of three sizes (fully expanded leaves in the middle of the canopy) was determined, and the leaves were subsequently immersed in water for 24–48 h to determine the water-saturated weight (SW), followed by 40 min of drying at 105 °C and 24 h of drying at 85 °C to determine the dry weight (DW). The RWC was calculated using the formula (FW-DW)/(SW-DW) × 100%. MDA content and CAT, POD, and SOD activities were measured using specific detection kits, following the manufacturer’s instructions (Nanjing Jiancheng Bioengineering Institute, Nanjing, China). Each experiment was repeated at least thrice.

### 2.3. Measurements of Stomatal Aperture

For microscopic observation of the stomata, a thin layer of nail polish was applied to the abaxial of the leaves of the ginkgo seedlings along the veins. The oil film with epidermal cells was stripped from the leaves using tweezers after being allowed to dry naturally for 5–10 min, and then observed under a LEICA DM500 microscope (Leica, Wetzlar, Germany). Stomatal apertures were examined using ImageJ 1.54 g software (National Institutes of Health, Bethesda, MD, USA). One plant was measured for each time point (D_0 d, D_15 d, D_22 d, D_Re3 d, CK_0 d, CK_15 d, CK_22 d, and CK_Re3 d). For each plant, two fully expanded leaves with similar sizes in the middle of the canopy were measured. Each standardized leaf was photographed from 5 different positions. More than 30 guard cells from each sample were measured to determine the stomatal aperture.

### 2.4. RNA Extraction, cDNA Library Construction, and RNA Sequencing

Total RNA was extracted from 100–200 mg of mature leaves (fully expanded leaves with similar sizes in the middle of the canopy) using a Mini Plant RNA Extraction Kit, following the manufacturer’s protocol (Vazyme, Nanjing, China). RNA quantity and quality were determined using a NanoDrop 2000 and Agilent 2100 Bioanalyzer (Thermo Fisher Scientific, Wilmington, DE, USA). cDNA libraries and RNA sequencing were performed using an Illumina Truseq^TM^ RNA Sample Prep Kit (Illumina, San Diego, CA, USA) at BIOZERON Bioinformatics Technology Co., Ltd. (Shanghai, China). Three cDNA libraries were generated from each sampling point and sequenced with three biological replicates.

### 2.5. Differentially Expressed Gene (DEG), Gene Ontology (GO), and Kyoto Encyclopedia of Genes and Genomes (KEGG) Analyses

Raw reads were filtered by removing adapters and low-quality sequences to obtain clean reads using Fastp (version 0.18.0) [28]. The high-quality clean reads were mapped to the *G. biloba* reference genome [29] via HISAT2 (v2.0.5) [30] with “-rna-strandness RF”. DEGs were identified based on transcripts per kilobase of exon model per million mapped reads (TPM) values with a log_2_ (fold change) ≥ 1 and adjusted *p*-value < 0.05 via the DESeq2 package [31]. Gene Ontology (GO) enrichment and Kyoto Encyclopedia of Genes and Genomes (KEGG) pathway analyses of the DEGs were performed using Cluster Profiler (3.4.4) software [32] with the parameters “pvalueCutoff = 0.05” and “qvalueCutoff = 0.05”.

### 2.6. Identification of Key Genes among the DEGs

To predict key pathway-related genes and TFs involved in the drought and re-watering responses of *G. biloba*, hmmsearch in the HMMER software (version 3.0) was used to align the ORFs to the hidden Markov model (HMM) profiles corresponding to pathway biosynthesis genes and TF protein domains downloaded from the Pfam protein family database. Heatmaps were generated using the TPM values with TBtools [33].

### 2.7. Quantitative Real-Time PCR (qRT-PCR) Analysis

An EasyScript^®^ First-Strand cDNA Synthesis SuperMix Kit was used for first-strand cDNA synthesized from total RNA, and qRT-PCR was performed with a TransStart^®^ Green qPCR SuperMix Kit (Transgene, Beijing, China) using a 7500 real-time PCR system (ABI, Los Angeles, CA, USA). The relative expression levels were determined via the 2^−ΔΔCt^ method. *GbGAPDH* was used as the endogenous control gene. All primers used in this study are listed in Supplementary Table S1.

## 3. Results

### 3.1. Variations in the Phenotypes and the Physiological and Biochemical Characteristics of G. biloba during Drought and Re-Watering

**Figure 1:**
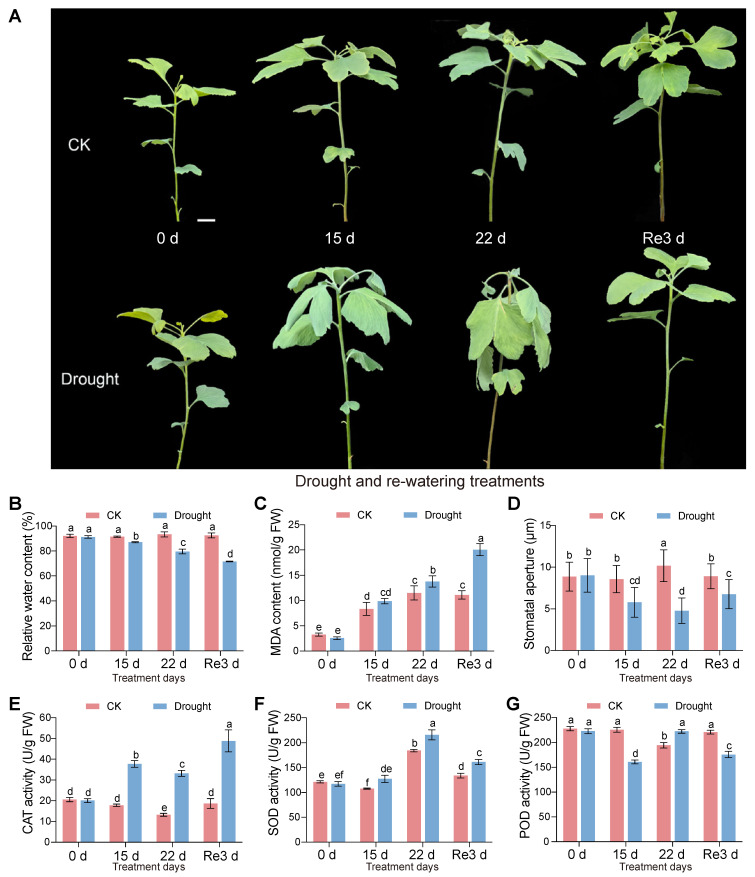
Phenotypes, physiological, and biochemical characteristics of *G. biloba* under drought and re-watering treatments. (**A**) Phenotypes of *G. biloba* drought for 0 d, 15 d, 22 d and re-watering 3 d, compared with the control group (CK) with regular irrigation. (**B**) Relative water content. (**C**) Malondialdehyde (MDA) content. (**D**) Stomatal aperture. (**E**) Catalase (CAT) activity. (**F**) Superoxide dismutase (SOD) activity. (**G**) Peroxidase (POD) activity. All data are presented as the mean ± SDs (n ≥ 3 biological replicates). Lowercase letters above bars indicate significant differences as determined by one-way ANOVA test followed by Tukey’s multiple comparisons test (*p* < 0.05).

All *G. biloba* seedlings showed good growth with blade stretching before the drought treatment (day 0). The ginkgo leaves of the drought group gradually wilted after 15 d and 22 d of drought treatment, accompanied by a gradual decrease in RWC, whereas the ginkgo leaves in CK maintained their stretched state and exhibited constant RWC (Figure 1A,B). Three days after re-watering (Re3 d), the leaves in the drought group returned to the same stretched shape as before treatment, with no significant difference from the control group; however, the RWC was not fully restored (Figure 1A,B). Compared with CK, regardless of whether they were at D_15 d, D_22 d, or D_Re3 d, the MDA content was significantly increased, and the stomatal aperture was significantly reduced (Figure 1C,D). Compared with CK, the activities of the antioxidant enzymes CAT and SOD were significantly increased at D_15 d, D_22 d, and D_Re3 d, but there was no significant difference before treatment (Figure 1E,F). POD activity at D_22 d was higher than that of CK_22d, whereas POD activity at D_15 d and D_Re3 d was lower than that of CK, indicating that POD may not have played a major role over these two periods (Figure 1G). Three days after re-watering, the phenotype of the ginkgo leaves had returned to the same level as that of CK, but the RWC, MDA content, stomatal aperture, and antioxidant enzyme activities had not returned to the same level (Figure 1). This may be because the biological processes and metabolic pathways in plants are not fully restored within a short period of re-watering after long-term drought stress.

### 3.2. Overall Analysis of the RNA-Seq Data

**Figure 2:**
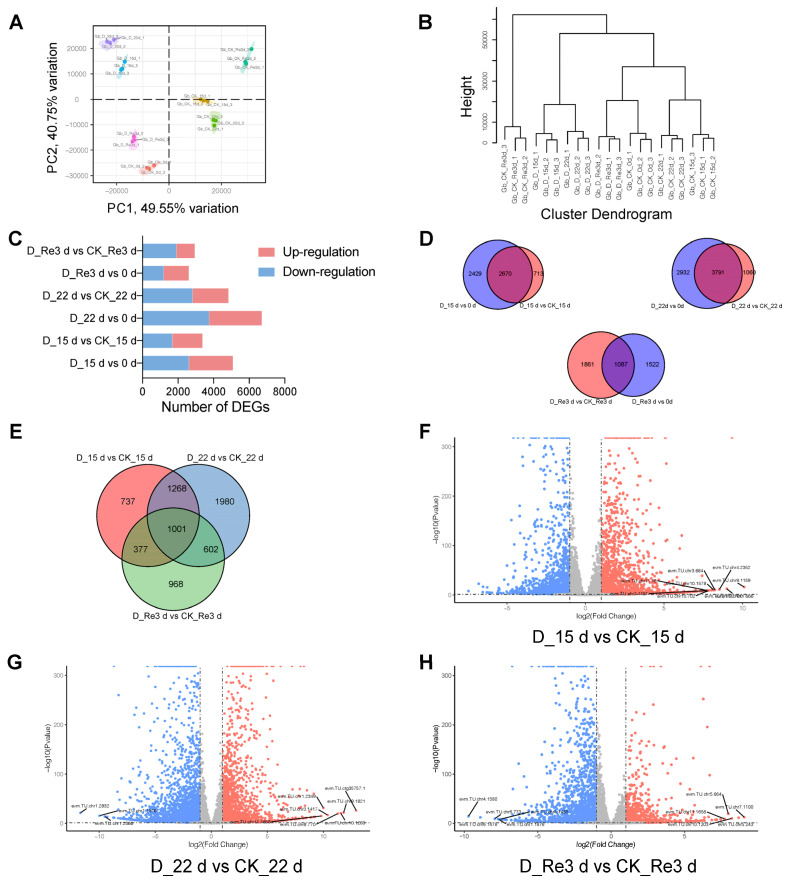
Identification and analysis of differentially expressed genes (DEGs) in *G. biloba* leaves during drought and re-watering treatments. (**A**) Principal component analysis of 21 samples based on transcripts per kilobase of exon model per million mapped reads (TPM). (**B**) Hierarchical clustering analysis of 21 samples based on Manhattan distance. (**C**) Number of DEGs in the drought and re-watering treatments. (**D**,**E**) Venn diagram of DEGs in the drought and re-watering treatments. (**F**–**H**) Volcano plots of DEGs at D_15 d vs. CK_15 d, D_22 d vs. CK_22 d, and D_Re3 d vs. CK_Re3 d. Red and blue dots indicate significantly upregulated and downregulated genes, respectively. The labeled genes indicate the most significantly differentially expressed genes.

To assess the gene expression patterns related to drought and re-watering in *G. biloba*, plants at four time points for the CK group (CK_0 d, CK_15 d, CK_22 d, and CK_Re3 d) and three time points (D_0 d was the same as CK_0 d) for the drought group (D_15 d, D_22 d, and D_Re3 d) were subjected to high-throughput RNA sequencing. A total of 21 cDNA libraries were generated from three biological replicates. High-throughput RNA-seq produced 40,036,090–51,058,130 raw reads from 21 cDNA libraries. After low-quality reads were filtered out, 40,006,012 to 51,019,792 clean reads were mapped to the reference genome, and the percentage of mapped reads per sample was very high, ranging from 96.74% to 98.33% (Table 1). The library of clean reads produced Q30 base percentages ranging from 95.28% to 96.09%, and the GC content ranged from 44.11 to 45.69% (Table 1). Based on the TPM data, the degree of intra- or intergroup variation was determined using principal component analysis (PCA). The samples collected at different time points in the drought and CK groups during the drought and re-watering treatments were grouped together with slight variability; nevertheless, there were significant differences between the groups, with up to 49.55% variance (Figure 2A). Hierarchical clustering analysis of the Manhattan distance and sample correlation analysis of the 21 samples were also performed. Three replicates of each sampling group clustered well (Figure 2B and Figure S2). In addition, the D_15 d and D_22 d branches clustered together, and the CK_15 d and CK_22 d branches clustered together. The D_Re3 d and CK_0 d branches also clustered together, indicating that gene expression levels in D_Re3 d were similar to those before treatment (Figure 2B). These results suggest that the sampling and RNA-seq data of *G. biloba* leaves during drought and re-watering in the present study are reliable for further analyses.

### 3.3. DEG Identification and Analysis in G. biloba Leaves during Drought and Re-Watering

A total of 2481, 2965, and 1407 genes were upregulated, whereas 2618, 3758, and 1202, genes were downregulated at D_15 d, D_22 d, and D_Re3 d, respectively, compared to day 0 (Figure 2C). Nevertheless, 1692, 2031, and 1038 genes were upregulated, whereas 1691, 2820, and 1910 genes were downregulated at D_15 d vs. CK_15 d, D_22 d vs. CK_22 d, and D_Re3 d vs. CK_Re3 d, respectively (Figure 2C). The number of DEGs in drought vs. CK was less than that in drought vs. 0 d, suggesting that the DEGs in drought vs. 0 d may be involved not only in the drought response but also in other developmental and metabolic pathways (Figure 2D). Among these DEGs, 2269 genes were common between D_15 d and CK_15 d and between D_22 d and CK_22 d (Figure 2E), suggesting that these genes are most likely to respond to drought stress in *G. biloba*. Of the 2269 genes, 1001 were common among D_15 d vs. CK_15 d, D_22 d vs. CK_22 d, and D_Re3 d vs. CK_Re3 d (Figure 2E), indicating that these genes were not only differentially expressed in the drought treatment but were also differentially expressed after a short period of re-watering, even though the phenotypes affected by drought stress were restored. Volcano plots display the upregulated, downregulated, and unregulated genes in *G. biloba* leaves at D_15 d vs. CK_15 d, D_22 d vs. CK_22 d, and D_Re3 d vs. CK_Re3 d in red, blue, and gray, respectively (Figure 2F–H). The labeled genes were the most significantly differentially expressed genes at each time point. evm.TU.chr1.1876, evm.TU.chr10.1203, and evm.TU.chr11.1658 were common among the most significantly differentially expressed genes at D_22 d vs. CK_22 d and D_Re3 d vs. CK_Re3 d. Notably, evm.TU.chr8.2086 was commonly upregulated at D_15 d vs. CK_15 d, D_22 d vs. CK_22 d, and D_Re3 d vs. CK_Re3 d, with a very high log_2_ (fold change) value (Figure S3), indicating that this gene is most likely positively related to drought and re-watering responses in *G. biloba.*

### 3.4. Functional Classification of the DEGs by GO and KEGG Pathway Analyses

**Figure 3:**
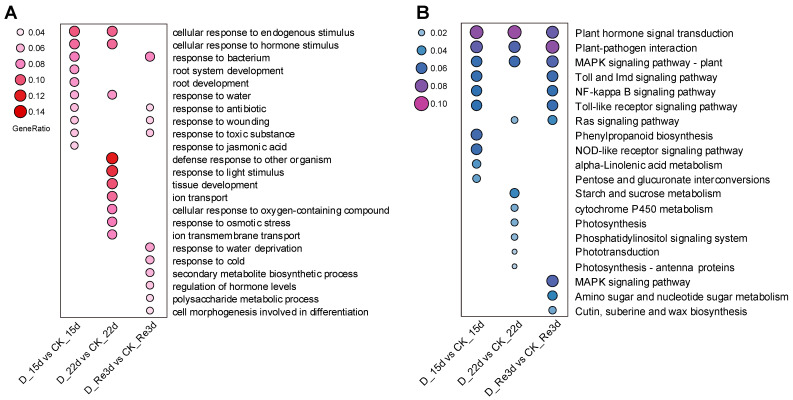
Gene Ontology (GO) and Kyoto Encyclopedia of Genes and Genomes (KEGG) pathway enrichment analyses of DEGs in the leaves of *G. biloba* under drought and re-watering treatments. (**A**) The top 10 GO enrichment items for each group. (**B**) The top 10 KEGG enrichment items for each group. D: drought; CK: control with regular irrigation.

To understand the major biological processes (BPs) induced by drought and re-watering, we conducted a GO enrichment analysis of DEGs at D_15 d vs. CK_15 d, D_22 d vs. CK_22 d, and D_Re3 d vs. CK_Re3 d. Cellular response to endogenous stimulus, cellular response to hormone stimulus, and response to water were the most enriched groups among the top 10 BPs at D_15 d vs. CK_15 d and D_22 d vs. CK_22 d, but these were not enriched at D_Re3 d vs. CK_Re3 d (Figure 3A). The cellular response to endogenous stimulus was the most highly enriched at D_15 d vs. CK_15 d. The defense response to other organisms was also most highly enriched at D_22 d vs. CK_22 d. We also found that four common GO terms were enriched in D_15 d vs. CK_15 d and D_Re3 d vs. CK_Re3 d, including responses to bacteria, antibiotics, wounding, and toxic substances (Figure 3A). However, no common GO terms were enriched in the D_22 d vs. CK_22 d or D_Re3 d vs. CK_Re3 d.

KEGG enrichment analysis was also conducted to explore the gene regulatory networks associated with drought and re-watering in *G. biloba*. The top 10 pathways for the most dominant DEGs in D_15 d vs. CK_15 d, D_22 d vs. CK_22 d, and D_Re3 d vs. CK_Re3 d are displayed. Three KEGG pathways, namely, plant hormone signal transduction, plant–pathogen interaction, and the plant MAPK signaling pathway, were enriched by the DEGs at D_15 d vs. CK_15 d, D_22 d vs. CK_22 d, and D_Re3 d vs. CK_Re3 d (Figure 3B). The DEGs in the above pathways reflected common responses to water stress in *G. biloba* during drought and re-watering treatments. Seven of the top ten pathways enriched in D_15 d vs. CK_15 d were different from those in D_22 d vs. CK_22 d, suggesting that the function of DEGs may differ with increased drought duration. In addition, the amino sugar and nucleotide sugar metabolism pathways and the cutin, suberine, and wax biosynthesis pathways were enriched only in D_Re3 d compared to CK_Re3 d (Figure 3B), suggesting that the DEGs involved in drought and re-watering were not entirely consistent in *G. biloba*.

### 3.5. Analyses of DEGs Involved in the Plant Hormone Signaling Pathway

**Figure 4:**
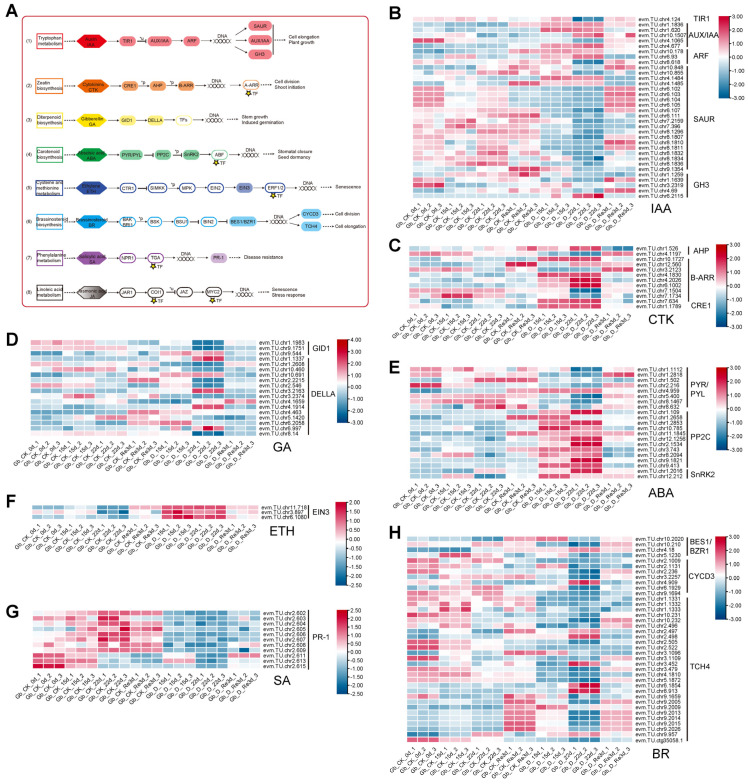
DEGs involved in plant hormone signal transduction during drought and re-watering treatments in *G. biloba*. (**A**) Schematic representation of the plant hormone signal transduction pathway. DEGs encoding key enzymes are shaded, and their expression is presented in the heatmap. The small, yellow five-pointed star indicates genes belonging to TFs. (**B**–**H**) Heatmap of DEGs encoding the auxin (IAA), cytokinin (CTK), gibberellin (GA), abscisic acid (ABA), ethylene (ETH), salicylic acid (SA), and brassinosteroid (BR) signal transduction pathways.

As shown in Figure 3B, the KEGG term “plant hormone signal transduction” was enriched at each time point, including D_15 d vs. CK_15 d, D_22 d vs. CK_22 d, and D_Re3 d vs. CK_Re3 d. To figure out the key DEGs involved in the plant hormone signal transduction pathway, including IAA, CTK, GA, ABA, ETH, BR, SA, and JA, a total of 36 gene families were identified. A total of 34 genes, comprising 1, 5, 3, 20, and 5 members, were linked to the transport inhibitor response 1 protein (TIR1), AUX/indoleacetic acid (IAA)-induced protein (AUX/IAA), auxin response factor (ARF), small auxin upregulated RNA (SAUR) family protein, and auxin-responsive glycoside hydrolase 3 (GH3) gene family in tryptophan metabolism for IAA signal transduction, respectively (Figure 4A,B). Most AUX/IAA and ARF genes were upregulated in the drought treatment compared to the CK, whereas most SAUR and GH3 genes were downregulated in the drought treatment (Figure 4B). The IAA signal transduction results suggest that drought stress induced AUX/IAA and ARF genes but repressed SAUR and GH3 genes. A total of 12 genes, comprising 2, 9, and 1 members, were linked to histidine-containing phosphotransferase (AHP), the two-component response regulator ARR-b family (B-ARR), and cytokinin receptor 1 (CRE1) in zeatin and diterpenoid biosynthesis for CTK signal transduction, respectively (Figure 4A,C). Eighteen structural genes, GIBBERELLIN-INSENSITIVE DWARF1 (GID1), and DELLA, involved in diterpenoid biosynthesis, were identified as DEGs related to the GA signal transduction pathway (Figure 4A,D). Similarly, carotenoid biosynthesis also had 21 genes linked to PYR/PYL, PP2C, and SnRK2, with 8, 11, and 2 genes, respectively, for ABA signal transduction (Figure 4A,E). Similarly, three ethylene-insensitive protein 3 (EIN3) involved in cysteine and methionine metabolism (Figure 4A,F) and 11 pathogenesis-related protein 1 (PR-1) involved in phenylalanine metabolism (Figure 4A,G) were identified as DEGs related to the ETH and SA pathways, respectively. In addition, 38 genes, comprising 4, 7, and 27 members, were linked to BRASSINAZOLE RESISTANT1 (BES1/BZR1), CYCLIN D3 (CYCD3), and xyloglucan:xyloglucosyl transferase TCH4 (TCH4) in brassinosteroid biosynthesis (Figure 4A,H) for BR signal transduction, respectively. Overall, these results suggest that ginkgo reprogrammed several phytohormones in response to drought stress.

### 3.6. Analyses of Differentially Expressed Transcription Factors Involved in the Drought and Re-Watering Response of G. biloba

**Figure 5:**
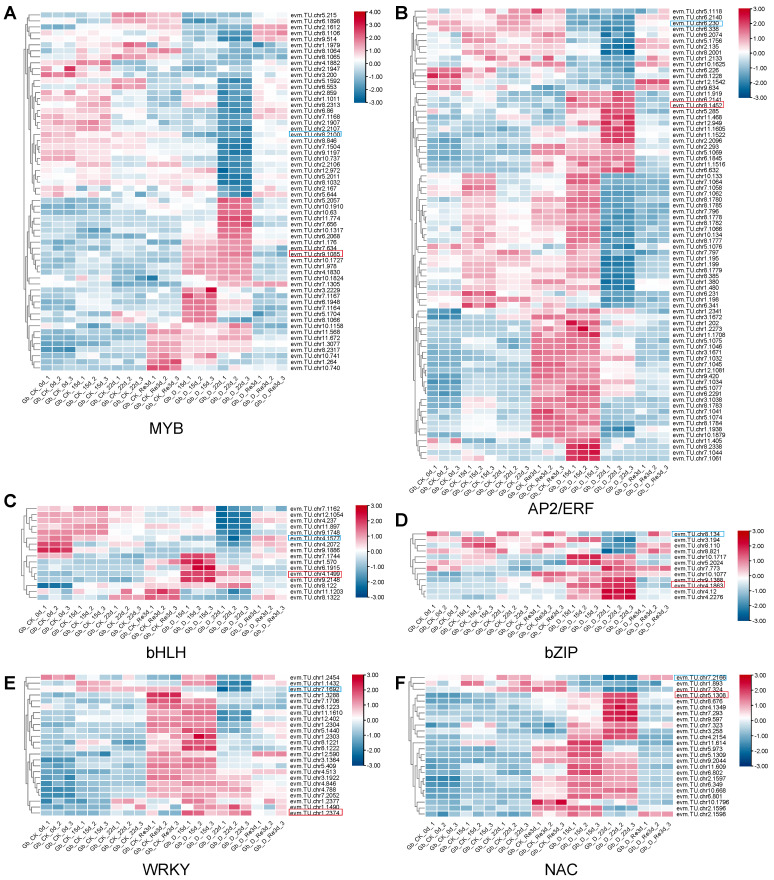
The major differentially expressed transcription factor families involved in the drought and re-watering treatments in *G. biloba*. (**A**) MYB. (**B**) AP2/ERF. (**C**) bHLH. (**D**) bZIP. (**E**) WRKY. (**F**) NAC. Genes labeled with red and blue rectangles indicate upregulation and downregulation, respectively.

TFs play essential roles in regulating physiological and biochemical processes in plants under stress conditions, including drought, by controlling the expression profiles of enzyme-encoding genes [6,26]. The six most common and crucial TFs were MYB, AP2/ERF, bHLH, NAC, WRKY, and bZIP, whose expression levels varied in *G. biloba* leaves during drought and re-watering (Figure 5). All TF families clustered into at least two groups, with one cluster positively correlated with drought stress and the other cluster negatively correlated with drought stress in *G. biloba*, implying that these families can either upregulate or downregulate the expression profiles of enzyme-encoding genes. Through expression pattern analysis, it was found that evm.TU.chr9.1085 and evm.TU.chr8.2100 were the MYBs most likely to be positively and negatively involved in the drought response of *G. biloba*, respectively (Figure 5A). Next, evm.TU.chr8.1452 and evm.TU.chr6.230 were the AP2/ERF genes most likely to be positively and negatively involved in drought response, respectively (Figure 5B). For bHLHs, evm.TU.chr4.1499 and evm.TU.chr4.1577 were most likely to be positively and negatively involved in drought response, respectively (Figure 5C). Meanwhile for bZIPs evm.TU.chr4.1863 and evm.TU.chr8.134 were most likely to be positively and negatively involved in drought response (Figure 5D). evm.TU.chr1.2374 and evm.TU.chr7.1692 were the WRKYs most likely to be positively and negatively involved in drought response, respectively (Figure 5E). Finally, evm.TU.chr5.1308 and evm.TU.chr7.2166 were the NACs most likely to be positively and negatively involved in the drought response of *G. biloba*, respectively (Figure 5F).

### 3.7. Expression Analysis of Key Genes Related to the Drought and Re-Watering Response of G. biloba by qRT-PCR

**Figure 6:**
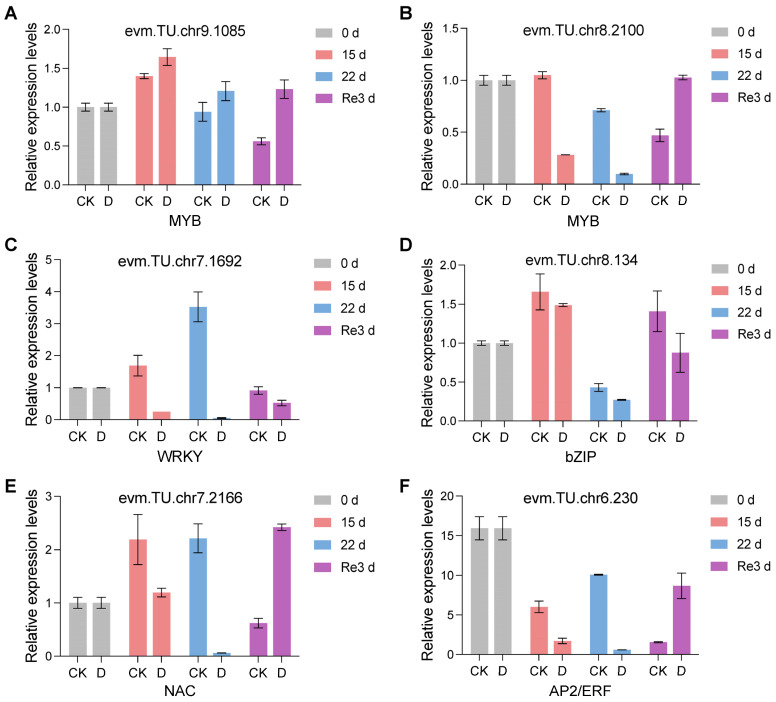
The relative expression levels of key differentially expressed transcription factors based on qRT-PCR analysis. (**A**) Upregulated MYB. (**B**) Downregulated MYB. (**C**) Downregulated WRKY. (**D**) Downregulated bZIP. (**E**) Downregulated NAC. (**F**) Downregulated AP2/ERF.

To validate the RNA-Seq results, six differentially expressed TFs were selected for qRT-PCR analysis. These results showed a similar trend to our RNA-seq results, providing further credence to their reliability (Figure 6). For instance, the expression of two MYB genes, evm.TU.chr9.1085 and evm.TU.chr8.2100, was upregulated and downregulated at D_15 d vs. CK_15 d and D_22 d vs. CK_22 d, respectively (Figure 6A,B), consistent with the heatmap shown in Figure 5A. Similarly, the expression of evm.TU.chr7.1692 (WRKY), evm.TU.chr8.134 (bZIP), evm.TU.chr7.2166 (NAC), and evm.TU.chr6.230 (AP2/ERF) was downregulated at D_15 d vs. CK_15 d and D_22 d vs. CK_22 d (Figure 6C–F), indicating that they are negative regulators of drought tolerance in *G. biloba.*

## 4. Discussion

### 4.1. Phenotypes, Physiological, and Biochemical Response during Drought Stress and Re-Watering in G. biloba

Drought stress usually causes a reversible decrease in leaf water content, membrane stability, and photosynthetic activity, resulting in increased ROS generation and membrane damage [2]. Several osmotic adjustment mechanisms are employed in plants under drought stress to increase the activities of enzymatic and non-enzymatic antioxidant systems, which enable plants to avoid damage from long-term drought stress and rapidly recover their physiological and biochemical functions after re-watering [34]. For example, during water stress, cells in the roots and leaves of *Medicago truncatula* plants display increased lipid peroxidation levels, proline content, and ROS content, whereas the leaves show reduced stomatal conductance and chlorophyll fluorescence. However, upon re-watering, *Medicago truncatula* plants recovered to levels similar to those under pre-stress control conditions [35]. In addition, after re-watering from drought stress, leaf water content, membrane stability, lipid peroxidation, photosynthetic processes, ROS accumulation, and antioxidative activities (SOD, POD, and CAT) fully recovered in moderately stressed wheat plants but did not completely recover in severely stressed ones [36].

We found that RWC in *G. biloba* leaves decreased continuously, while MDA content increased continuously during drought stress and re-watering (Figure 1B,C), implying that drought stress induced ROS accumulation and could not be eliminated after 3 d of re-watering, even though the severely wilted phenotypes of drought-stressed plants were rapidly restored. The stomatal aperture decreased continuously during drought stress but significantly increased after re-watering, although it was still smaller than that of CK (Figure 1D). Correspondingly, the increased activities of antioxidant enzymes under drought stress and re-watering, including CAT and SOD (Figure 1E,F), imply that these enzymes may continuously alleviate ROS damage, even if the drought stress is removed. These results are similar to those of other plants, including wheat, licorice, and *Medicago truncatula* under drought stress and re-watering [35,36,37], suggesting that the ability to maintain functions of some antioxidant enzymes during drought and short-term re-watering is essential for *G. biloba* to sustain productivity and development during natural drought disasters.

In this study, as the drought duration increased from 15 d to 22 d, the leaves of *G. biloba* gradually wilted (Figure 1A) and the number of DEGs also gradually increased (Figure 2C), which means that increased genes are needed for *G. biloba* to combat increasingly long-term water shortages. Even if the leaves were severely wilted after a long-term (22 d) drought, it only took 3 days after re-watering for the leaves of *G. biloba* to return to the same state before the drought treatment (Figure 1A), and the number of DEGs decreased (Figure 2C). There were 1001 DEGs between the three groups (D_15 d vs. CK_15 d, D_22 d vs. CK_22 d, and D_Re3 d vs. CK_Re3 d) that were the same (Figure 2E), which suggests that these DEGs not only maintained their differential expression in long-term drought but also after short-term re-watering. These genes may be related to ginkgo’s high resistance to long-term drought.

### 4.2. Plant Hormone Signaling Was Associated with Drought Stress and Re-Watering in G. biloba

Phytohormones are signaling compounds that control essential aspects of growth, development, and various stress responses in plants. Among the eight well-known plant hormones (IAA, CTK, GA, ABA, ETH, BR, SA, and JA), ABA is a central integrator that activates adaptive signaling cascades and hormonal crosstalk during the drought stress response in plants [8]. Drought stress is first induced in the roots, followed by the induction of ABA biosynthesis in the shoots via hydraulic signals and CLE25 peptide-mediated induction of NCED3 expression. The ABA-signaling pathway involves the recognition of ABA by PYR/PYL receptors, inactivation of PP2C, and subsequent activation of SnRK2s [38]. When whole *Arabidopsis thaliana* plants were exposed to drought stress, the endogenous ABA levels increased rapidly, followed by inducing stomatal closure in response to drought stress [39]. Additionally, drought stress increases the expression of transcriptional repressors of auxin response, including *IAA5* and *IAA19*. Mutations in AUXs/IAAs reduce the survival rate of plants under drought stress [40]. Moreover, drought stress reduces CTK content and signaling via type-A and type-B ARRs that interact with SnRK2s, resulting in increased ABA sensitivity [41]. Similarly, GA signaling interferes with ABA signaling via DELLA protein interactions with the ABA-related TFs ABF2 and BR signaling via BR and ABA crosstalk at the level of BES1 and RD26 mediated transcriptional regulation under drought stress conditions [8].

Plant hormone signal transduction was enriched by the DEGs during both drought and re-watering, including at D_15 d vs. CK_15 d, D_22 d vs. CK_22 d, and D_Re3 d vs. CK_Re3 d (Figure 3B), suggesting the involvement of ABA and other hormone signaling pathways in the adaptive response of *G. biloba* to drought conditions and recovery from stress. Distinct expression patterns of many DEGs involved in plant hormone signaling, including 36 gene families, such as PYR/PYLs, SnRK2, IAAs, and ARFs, were observed in both drought stress and re-watering (Figure 4), suggesting that plant hormone signaling pathways are likely associated with these responses in *G. biloba*. These results are similar to those reported for other plants [42], which showed that plant signaling pathway-associated genes were enriched and predicted to be involved in the drought response. However, we did not identify DEGs involved in JA signal transduction (Figure 4), suggesting that JA signaling pathways may not be associated with the drought and re-watering responses in *G. biloba*, which is different from other species.

### 4.3. TFs May Play Crucial Roles in the Response to Drought Stress and Re-Watering in the Leaves of G. biloba

Many TFs families, such as MYB, bHLH, AP2/ERF, NAC, WRKY, and bZIP, play important roles in the abiotic stress response of plants, including drought stress, by controlling the expression of downstream biosynthetic enzyme-encoding and stress-responsive genes [43]. For example, overexpression of DREB1/CBF (a subfamily of AP2/ERF TFs) in different plants, including tomato, potato, tobacco, rice, soybean, and wheat, resulted in increased expression of several stress-responsive genes and improved tolerance to drought [43]. In addition, the overexpression of *AtNAC019/055/072* [25] or *AtWRKY18/40/60* [9], or *AtMYB44*/*60*/*96* [44] in *Arabidopsis* conferred drought stress tolerance in an ABA-dependent manner. Similarly, overexpression of stress-responsive NAC1 (SNAC1) in rice conferred tolerance to severe drought stress without phenotypic and yield changes, and overexpression of *OsNAC6* in rice resulted in improved water retention by controlling stomatal closure under dehydration stress [45]. Recent studies have also determined the roles of bZIP TFs, such as *OsbZIP2*, *OsbZIP23*, *OsbZIP42*, and *OsbZIP46*, in rice, in positively regulating ABA-dependent gene expression and drought stress responses [46].

In this study, we identified six TF families that may play crucial roles in the response to drought stress and re-watering in the leaves of *G. biloba*. The MYB, bHLH, AP2/ERF, NAC, WRKY, and bZIP TF genes displayed in the heatmap (Figure 5) were upregulated or downregulated in response to drought stress and re-watering, suggesting that they are respectively positive or negative regulators of drought stress in *G. biloba*. The expression patterns from the qRT-PCR results for the selected six TFs showed a similar trend to our RNA-seq results (Figure 6), providing more evidence for the candidate TFs. Further transgenic validation experiments are needed to reveal their function and regulatory roles in the response to drought stress and re-watering in *G. biloba*.

## 5. Conclusions

Insights were gained into the physiological and molecular responses to drought stress and re-watering in *G. biloba* by performing physiological and transcriptomic analyses of drought treatment for 15 d and 22 d and re-watering for 3 d. Pathways such as plant hormone signal transduction, plant–pathogen interaction, and the plant MAPK signaling pathway were identified as commonly enriched KEGG pathways involved in the response to drought stress and re-watering. Moreover, several DEGs displayed unique enrichment patterns between drought stress and re-watering, suggesting that they were associated with either drought stress or re-watering. Furthermore, the expression patterns of DEGs involved in the plant hormone signal transduction pathway were determined, and TFs such as WRKYs, MYB, bHLH, AP2/ERF, NAC, and bZIP were identified, providing further evidence for identifying key candidate genes for improving the drought tolerance of *G. biloba* and other forest species.

## Figures and Tables

**Table 1:** Overview of the RNA-sequencing data and quality control checks of 21 cDNA libraries of *G. biloba* leaves.

Sample	Total Reads	Clean Reads	Total Mapped %	Q30 %	GC %
Gb_CK_0d_1	48,222,976	48,186,740	97.57	95.77	44.52
Gb_CK_0d_2	43,988,902	43,956,016	97.78	95.72	44.68
Gb_CK_0d_3	51,044,520	51,006,222	97.74	95.50	44.69
Gb_CK_15d_1	41,067,644	41,037,102	97.39	95.69	44.91
Gb_CK_15d_2	42,759,844	42,728,290	97.35	95.72	45.06
Gb_CK_15d_3	42,170,744	42,139,574	97.26	95.63	45.07
Gb_CK_22d_1	42,203,262	42,171,764	97.46	95.28	45.69
Gb_CK_22d_2	43,104,858	43,072,790	97.52	95.98	45.53
Gb_CK_22d_3	43,082,162	43,050,272	97.51	95.48	45.65
Gb_CK_Re3d_1	41,319,086	41,287,526	96.77	96.00	45.39
Gb_CK_Re3d_2	40,531,662	40,501,260	96.76	95.59	45.28
Gb_CK_Re3d_3	46,754,752	46,719,608	96.74	95.88	45.06
Gb_D_15d_1	46,136,970	46,102,948	98.22	96.00	44.38
Gb_D_15d_2	44,568,560	44,534,584	98.25	96.09	44.30
Gb_D_15d_3	51,058,130	51,019,792	98.25	95.97	44.36
Gb_D_22d_1	40,036,090	40,006,012	98.32	95.61	43.89
Gb_D_22d_2	40,427,736	40,397,182	98.33	95.83	44.16
Gb_D_22d_3	41,467,182	41,437,562	98.33	95.76	44.11
Gb_D_Re3d_1	43,216,190	43,183,472	97.97	95.69	44.47
Gb_D_Re3d_2	40,386,156	40,355,568	98.03	96.06	44.35
Gb_D_Re3d_3	40,377,458	40,347,646	98.01	95.86	44.55

## Data Availability

The raw sequence data reported in this paper have been deposited in the Genome Sequence Archive (Genomics, Proteomics & Bioinformatics 2021) in the National Genomics Data Center (Nucleic Acids Res 2022), China National Center for Bioinformation/Beijing Institute of Genomics, Chinese Academy of Sciences (GSA: CRA018280), and are publicly accessible at https://ngdc.cncb.ac.cn/gsa. Accessed on 20 September 2024.

## Supplementary Materials

The following supporting information can be downloaded at: https://www.mdpi.com/article/10.3390/plants13192685/s1, Figure S1. Schematic diagram of *G. biloba* seedlings under drought and re-watering treatments. Figure S2. Heatmap of all 21 samples based on the sample correlation analysis. Figure S3. The expression pattern analysis of the DEG evm.TU.chr8.2086. Table S1. All primers used in the current study.
