# Supplementary material for: Transcriptome Profiling Identifies Plant Hormone Signaling Pathway-Related Genes and Transcription Factors in the Drought and Re-Watering Response of Ginkgo biloba
Source: Plants (Basel). 2024 Sep 25;13(19):2685. doi: 10.3390/plants13192685 (PMC11478988; doi:10.3390/plants13192685)
Supplement: Supplementary file 1 [file plants-13-02685-s001.zip › Supplementary Figures.pptx]

## Slide 1
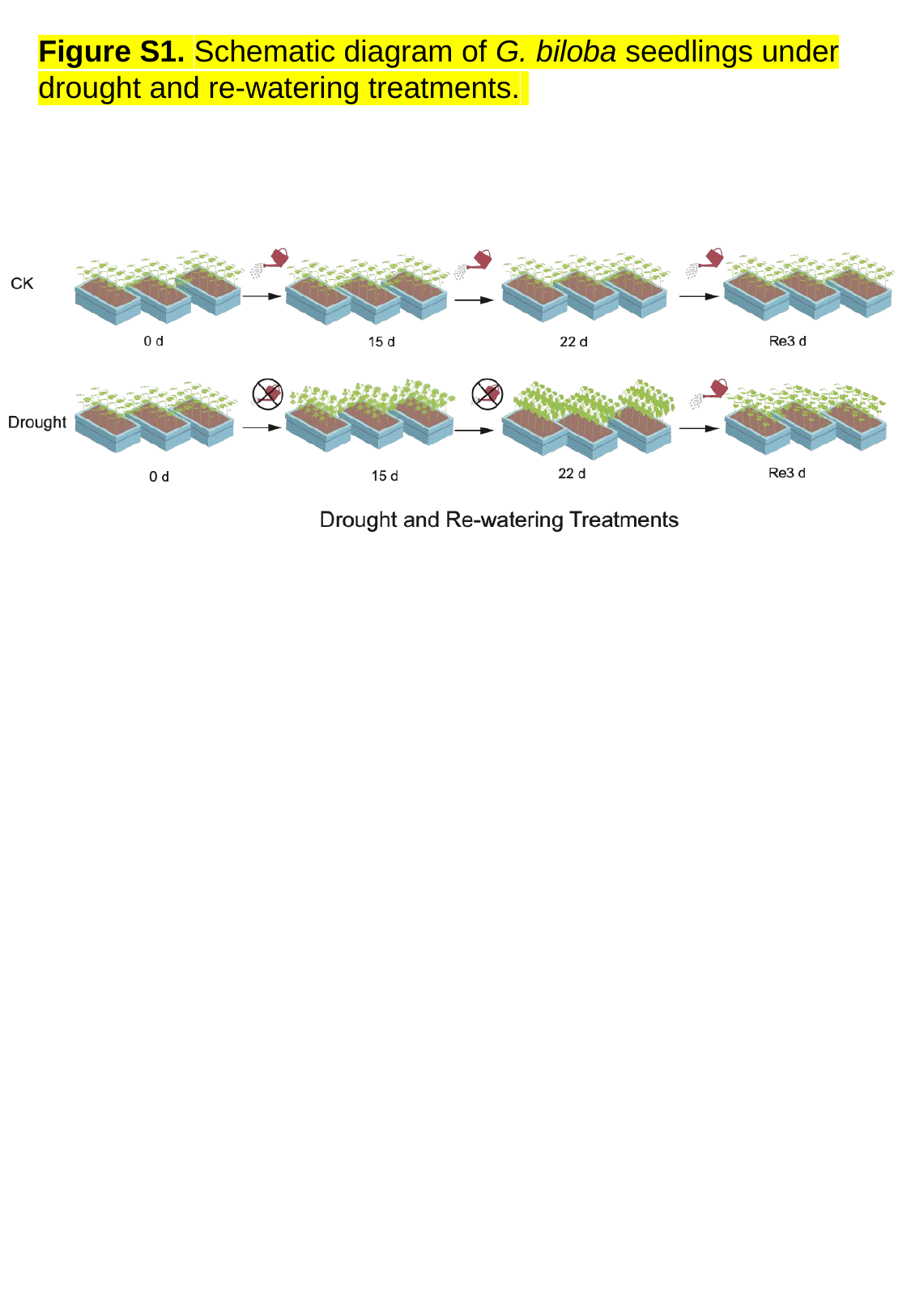

Figure S1. Schematic diagram of G. biloba seedlings under drought and re-watering treatments.

## Slide 2
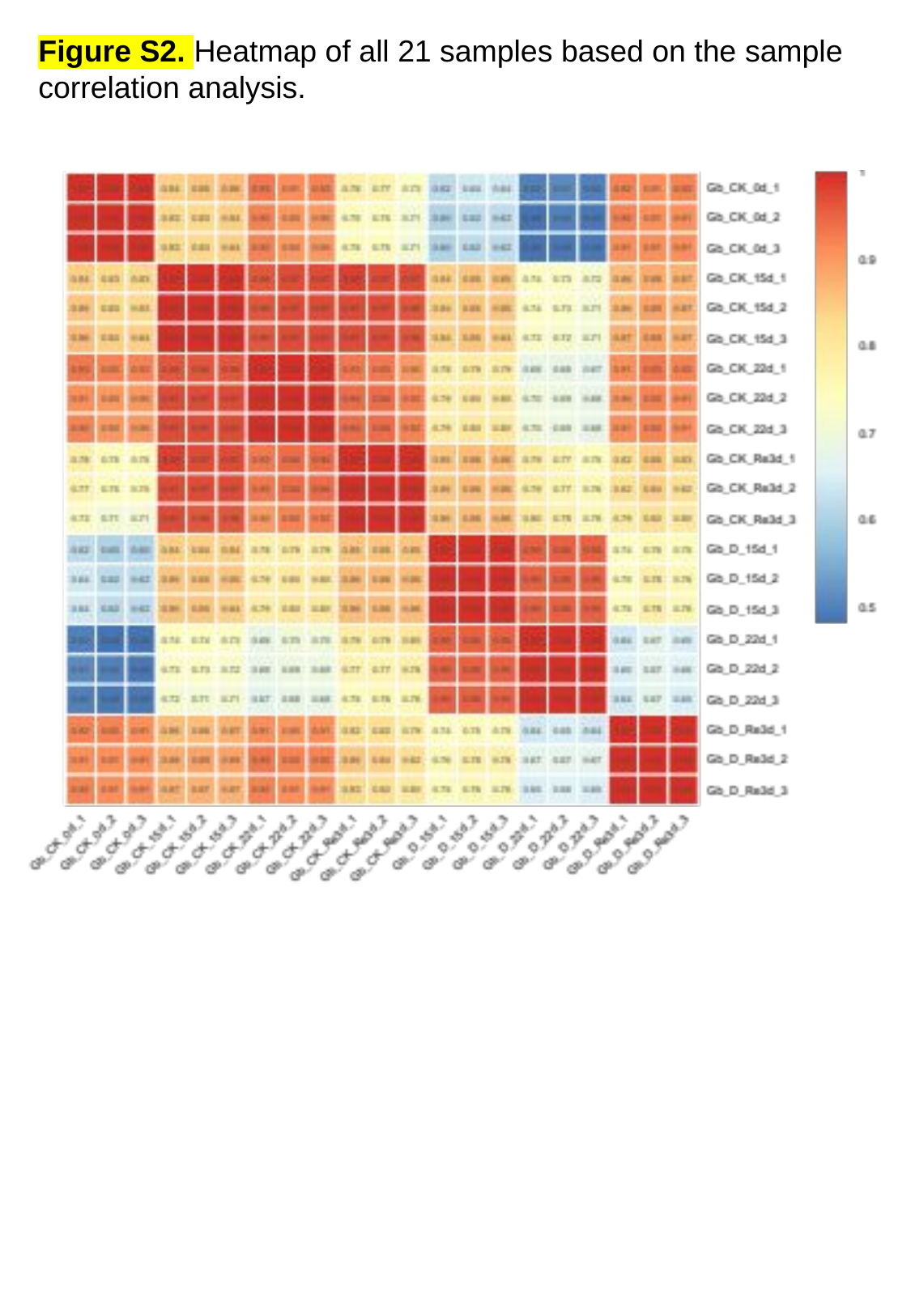

Figure S2. Heatmap of all 21 samples based on the sample correlation analysis.

## Slide 3
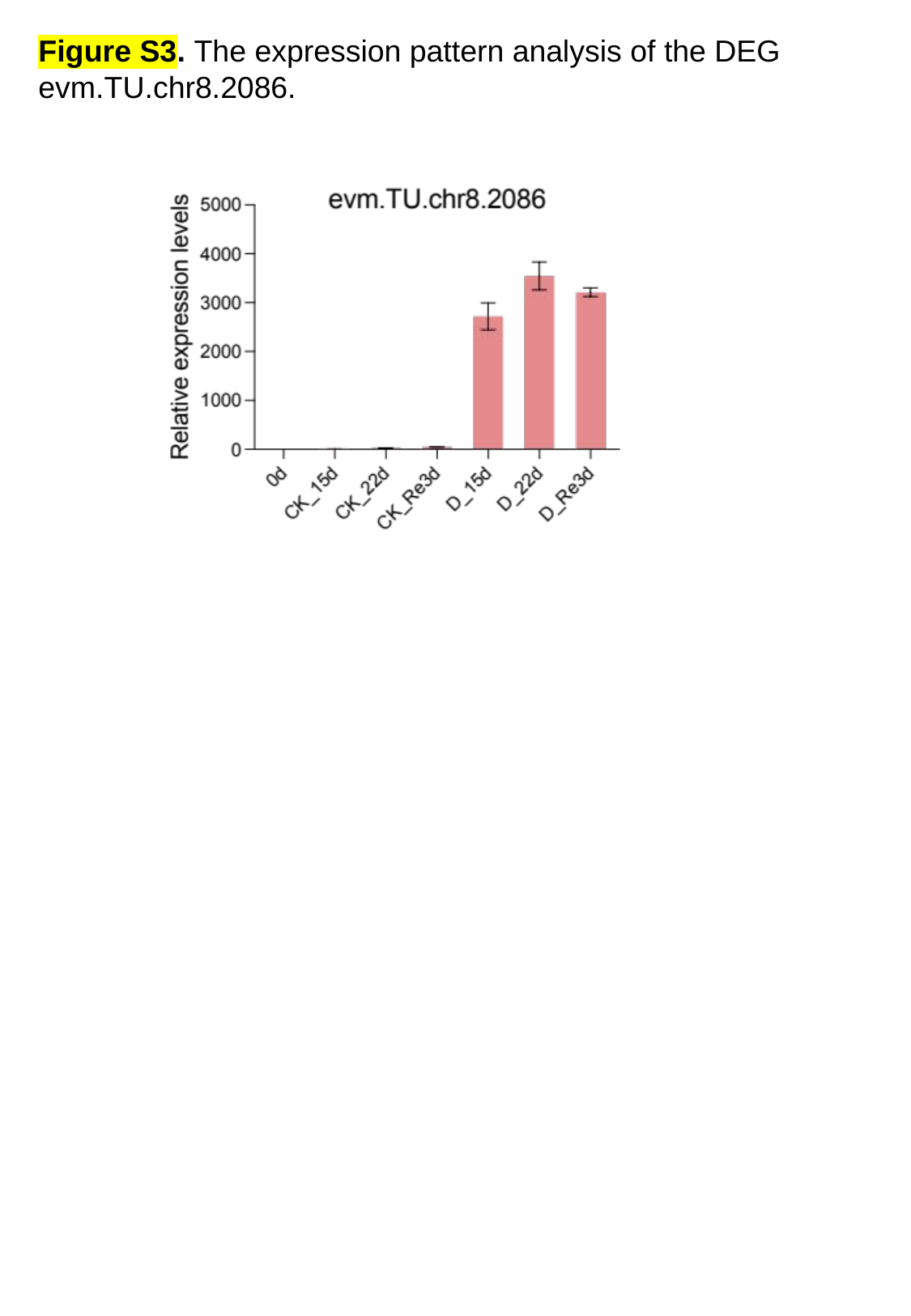

Figure S3. The expression pattern analysis of the DEG evm.TU.chr8.2086.
